# Fine tuning of cytosolic Ca ^2+^ oscillations

**DOI:** 10.12688/f1000research.8438.1

**Published:** 2016-08-19

**Authors:** Geneviève Dupont, Laurent Combettes

**Affiliations:** 1 Unité de Chronobiologie Théorique, Faculté des Sciences, Université Libre de Bruxelles (ULB), Brussels, Belgium; 2 Interactions Cellulaires et Physiopathologie Hépatique, UMR-S 1174, Université Paris Sud, Orsay, France

**Keywords:** calcium oscillations, store-operated calcium entry, mitochondrial Ca2+ uniporter, mitochondrial Ca2+ uptake 1

## Abstract

Ca ^2+^ oscillations, a widespread mode of cell signaling, were reported in non-excitable cells for the first time more than 25 years ago. Their fundamental mechanism, based on the periodic Ca ^2+^ exchange between the endoplasmic reticulum and the cytoplasm, has been well characterized. However, how the kinetics of cytosolic Ca ^2+^ changes are related to the extent of a physiological response remains poorly understood. Here, we review data suggesting that the downstream targets of Ca ^2+^ are controlled not only by the frequency of Ca ^2+^ oscillations but also by the detailed characteristics of the oscillations, such as their duration, shape, or baseline level. Involvement of non-endoplasmic reticulum Ca ^2+^ stores, mainly mitochondria and the extracellular medium, participates in this fine tuning of Ca ^2+^ oscillations. The main characteristics of the Ca ^2+^ exchange fluxes with these compartments are also reviewed.

## Introduction

**Figure 1.**
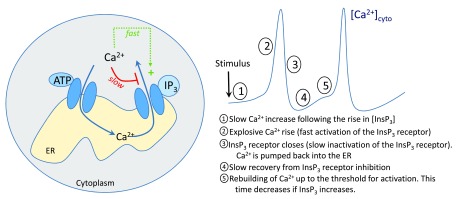
Basic mechanism of cytosolic Ca ^2+^ oscillations in non-excitable cells. These oscillations are initiated by the stimulus-induced rise in inositol 1,4,5-trisphosphate (InsP _3_) concentration and occur through a repetitive exchange of Ca ^2+^ between the cytoplasm and the endoplasmic reticulum (ER).

Most of the time, the hormone-induced Ca ^2+^ increases that activate a variety of essential intracellular processes take the form of Ca ^2+^ oscillations. In non-excitable cells, these repetitive spikes mainly arise through the periodic exchange of Ca ^2+^ between the endoplasmic reticulum (ER) and the cytosol, through the interplay between inositol 1,4,5-trisphosphate (InsP _3_)-sensitive Ca ^2+^ channels and SERCA pumps ^1, 2^. This basic mechanism, summarized in Figure 1, has now been well characterized and accounts for the observed increase in the frequency of Ca ^2+^ oscillations with increasing concentrations of InsP _3_ accompanying the rise in stimulation. Such a process is referred to as *frequency encoding*. It was often hypothesized that oscillations provide a digital signal to downstream effectors that are in turn stimulated in an ON or OFF manner. Indeed, if a process is activated above a threshold Ca ^2+^ concentration, oscillations allow Ca ^2+^ to reach this threshold repetitively even if the average Ca ^2+^ signal remains below the threshold ^3, 4^.

Based on the observed frequency encoding of the extracellular signal, it was also postulated that the physiological response in the form of secretion, gene expression, proliferation, etc., would in turn be sensitive to the frequency of Ca ^2+^ oscillations ^5– 8^. Although intuitively attractive, such *frequency sensitivity* of the downstream targets of Ca ^2+^ has not been well corroborated by data. Besides the beautiful example of Ca ^2+^-dependent calmodulin kinase II regulation by high-frequency Ca ^2+^ spikes ^9^ or of selective gene expression in T-lymphocytes ^4^, there are few clear examples of physiological responses to Ca ^2+^ increases that are quantitatively controlled by the frequency of the Ca ^2+^ spikes. This statement does not mean that frequency encoding does not occur or that the frequency of Ca ^2+^ oscillations does not affect the extent of the Ca ^2+^-mediated physiological response. Indeed, a higher frequency of oscillations implies a larger average Ca ^2+^ level, which may be *per se* the reason for the larger response. However, modulating the amplitude of the oscillations, their baseline level, or the duration of the spikes also modifies the average level and hence the response. As another example, spikes preceded by an important pacemaker-like Ca ^2+^ increase could activate slower downstream targets characterized by a low threshold of activation. In such cases, frequency cannot be considered as the key characteristic of the oscillatory pattern and the response is not simply frequency sensitive. However, in the numerous studies about Ca ^2+^ oscillations, frequency is the most studied parameter and the most commonly related to the extent of Ca ^2+^-mediated physiological responses.

**Figure 2.**
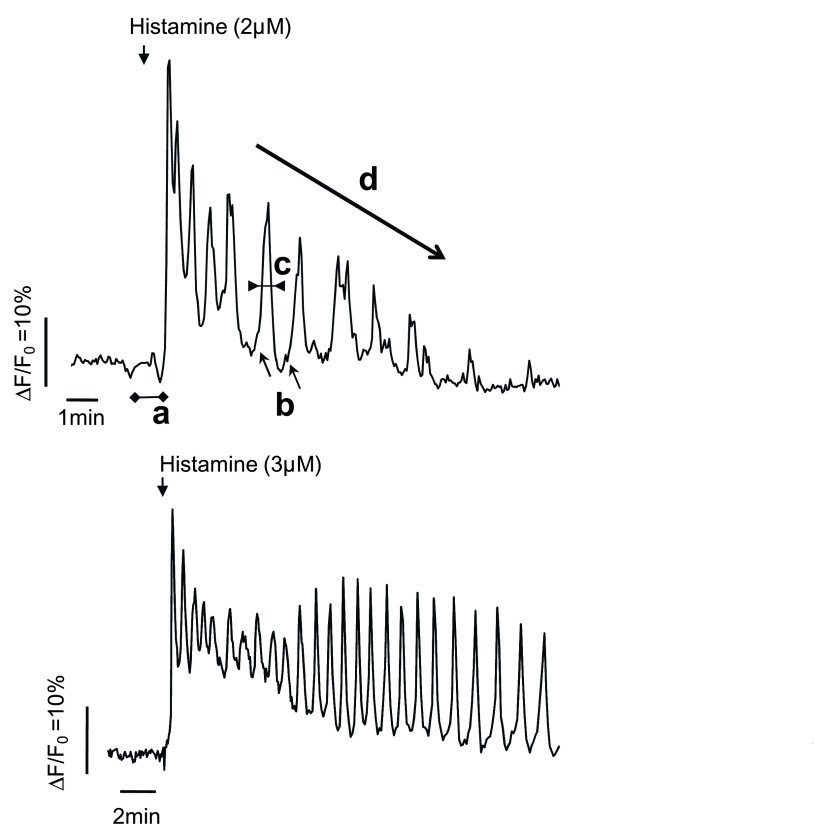
Various characteristics of Ca ^2+^ oscillations that participate in fine tuning. Traces show typical curves of Fluo4 loaded HeLa cells challenged with either 2 µM histamine (upper trace) or 3 µM (lower trace). Calcium imaging was performed as described previously ^10^. Fluorescence images were collected every 3 seconds by an EM-CCD camera (Hamamatsu), digitized, and integrated in real time by an image processor (Metafluor). Letters indicate characteristics of Ca ^2+^ oscillations that, besides their frequency, can affect the cellular response to these repetitive Ca ^2+^ increases (a: latency of the Ca ^2+^ response to the stimulation, b: minimal Ca ^2+^ level between the spikes or baseline Ca ^2+^, c: duration, or half-width, of the spikes, and d: rate of decrease of the response or degree of sustainability).

In fact, the relative scarcity of phenomena that are purely controlled by the frequency of Ca ^2+^ oscillations is not so surprising given that the period of Ca ^2+^ oscillations can be subject to a significant level of randomness ( Figure 2 and 8, 10). In some instances, it has even been explicitly observed that the frequency does not by itself regulate the extent of the second-messenger-mediated response. This is the case, for example, for carbachol-induced salivary secretion by acinar cells ^11^. At mammalian fertilization, the total integrated Ca ^2+^ signal input is the most relevant parameter ensuring completion of fertilization-associated events ^12^. Interestingly, frequency encoding is also not a universal feature of Ca ^2+^ oscillations, as it was shown in some cases, such as in acetylcholine-stimulated pancreatic acinar cells ^13^, methacholine-stimulated lacrimal cells ^14^, fish hepatocytes ^15^, or in cell lines expressing the metabotropic glutamate receptor 5 ^16^, that an increase in stimulation does not affect the frequency of the resulting Ca ^2+^ oscillations. In these cases, of course, it cannot be expected that the frequency of Ca ^2+^ oscillations would be the way by which cells encode the information related to the level of response that is precisely triggered by the stimulation.

Also, recent investigations tend to suggest that rather than the frequency alone, the detailed dynamic characteristics of the Ca ^2+^ increase pattern play an important role in determining the extent of the cell response. As illustrated in Figure 2, in addition to frequency, Ca ^2+^ oscillations can vary in the amplitude and the width of the spikes, the baseline Ca ^2+^ level, and the degree of sustainability. We refer to modifications of one of these characteristics as *fine tuning of Ca ^2+^ oscillations* to emphasize that they imply fine regulation of cytosolic Ca ^2+^ that goes behind the mechanism schematized in Figure 1 accounting for the existence and the frequency of oscillations. Various observations corroborate that these properties are important determinants for the physiological response of the cell. For example, the CD147 factor promotes oncogenic activities and influences the progression of hepatocellular carcinoma by enhancing both the amplitude and the frequency of ER-dependent Ca ^2+^ oscillations ^17^. In intestinal stem cells, dietary and stress stimuli are integrated in such a way that frequent and robust Ca ^2+^ oscillations are associated with a poised proliferative state, while smoother oscillations on a more elevated level accompany active proliferation ^18^. Fine tuning of Ca ^2+^ signals also plays a role in the differentiation of neuronal and muscle cells (see 19 for review). In astrocytes, knocking-down the Na ^+^–Ca ^2+^ exchanger (NCLX) that mediates Ca ^2+^ release from mitochondria slightly affects cytosolic Ca ^2+^ changes and, by doing so, significantly reduces Ca ^2+^-dependent processes, such as glutamate release, wound closure, and proliferation ^20^. Cell survival, death, and adaptation are sensitive to changes in Ca ^2+^ patterns due to the interplay between ER/cytoplasmic Ca ^2+^ exchanges and mitochondria and lysosomes ^21^. *Shigella* bacteria also fine tune the Ca ^2+^ responses when they invade epithelial cells. While the wild-type strain induces rather smooth and low-amplitude Ca ^2+^ variations in the cytoplasm of the host cell, a less-invasive mutant strain induces more robust Ca ^2+^ responses which, paradoxically, are associated with a higher survival of the host cells during the first hours following invasion ^22^.

On another level, the precise isoforms of InsP _3_ receptors expressed by a given cell – which have been shown to substantially affect the shape of the Ca ^2+^ oscillations ^23– 25^ – are critical for cell death and survival decisions ^26^. Finally, bioinformatics analyses highlighted that in cancer cells and tissues, the main processes associated with Ca ^2+^ dynamics that are perturbed are the mechanisms of store-operated calcium entry (SOCE) and of calcium reuptake into mitochondria ^27^. Both of these processes are related to the fine tuning of Ca ^2+^ oscillations, as discussed below. Altogether, these observations call for a more detailed understanding of oscillation-associated Ca ^2+^ dynamics. Understanding why Ca ^2+^ oscillates and what regulates the frequency of oscillations is not sufficient to understand their physiological impact, but the duration and shape of the peaks, their sustainability, and the baseline Ca ^2+^ level must be carefully taken into account. In the following sections, we elaborate on two key controllers of the InsP _3_R-based Ca ^2+^ oscillations, both related to Ca ^2+^ stores other than the ER, namely the mitochondria and the extracellular medium. We briefly review and discuss some of the main recent observations about their interplay with the InsP _3_-induced Ca ^2+^ spikes.

## Mitochondrial Ca ^2+^ uptake and release

**Figure 3.**
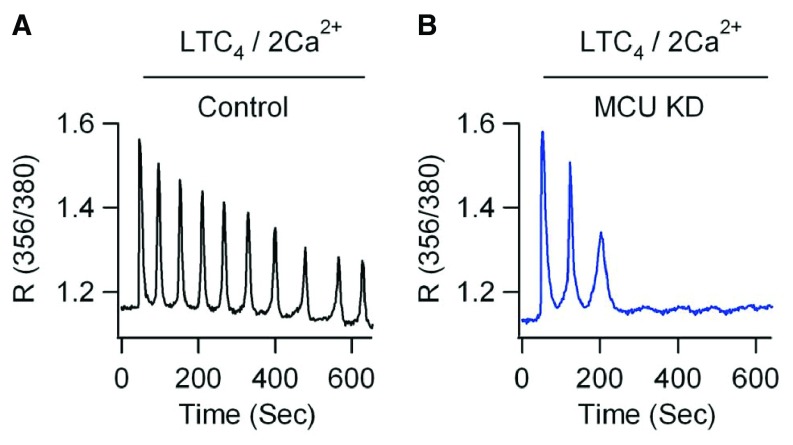
The mitochondrial Ca ^2+^ uniporter (MCU) participates in the fine tuning of Ca ^2+^ oscillations. **A.** Control recording of Ca ^2+^ responses in rat basophilic leukemia (RBL)-1 cells stimulated by LTC4 in 2 mM external Ca ^2+^. **B.** Same recording after MCU knockdown. Ca ^2+^ entry in mitochondria via the MCU broadens cytosolic Ca ^2+^ spikes and decreases their sustainability. From Samanta *et al*. ^37^. Shown are the ratios of fluorescence of Fura-2 loaded cells excited at 356 and 380 nm.

By stimulating the activity of key enzymes involved in mitochondrial ATP synthesis, Ca ^2+^ entry into mitochondria stimulates metabolism, thereby coupling ATP synthesis with energy demand ^28^. That Ca ^2+^ exchange between the cytosol and the mitochondria in turn affects InsP _3_-induced cytosolic Ca ^2+^ signals was put forward quite early ^29, 30^, but this concept was somewhat put aside for a decade. The molecular identification of the mitochondrial Ca ^2+^ uniporter (MCU), a voltage, cytosolic, and mitochondrial Ca ^2+^-sensitive transporter ^31– 34^, awakened interest in this question. Ca ^2+^ entry into mitochondria through the MCU is a highly non-linear function of cytosolic Ca ^2+^ ^33^. The MCU is in fact the Ca ^2+^ pore-forming component of the uniporter and is part of a large complex of proteins that are required for Ca ^2+^ channel activity or to regulate it under various conditions. For example, MICU1 (mitochondrial Ca ^2+^ uptake 1) limits mitochondrial Ca ^2+^ influx at low cytosolic Ca ^2+^ concentration and the interaction between the MCU and MICU1 requires the expression of another component, called EMRE for essential MCU regulator ^34, 35^. Ca ^2+^ efflux back into the cytoplasm occurs through a NCLX. As expected, modifying any of these pathways affects the frequency of the oscillations; interestingly, increasing the activity of the MCU can both increase and decrease the frequency of oscillations ^36^. In addition to its effect on the frequency of the oscillations, the MCU controls the width of the spikes and the sustainability of the oscillations, as knocking-down the MCU broadens Ca ^2+^ oscillations and accelerates the rundown of the oscillations in rat basophilic leukemia (RBL)-1 cells ( Figure 3). Such rundown suppresses gene expression in response to leukotriene receptor activation ^37^. Mitochondria also affect the rate of rise and fall of cytosolic Ca ^2+^ and thus the half-width and duration of the spike. More specifically, mitochondria smooth out cytosolic Ca ^2+^ changes mainly because they have a ~30 times larger Ca ^2+^ buffering capacity than the cytoplasm ^38^. Also, because of their slow dynamics, mitochondria continue releasing Ca ^2+^ between subsequent releases of Ca ^2+^ from the ER, thus playing a key role in determining the baseline cytosolic Ca ^2+^ level. Thus, mitochondrial Ca ^2+^ handling through the MCU and the NCLX clearly fine tunes cytosolic Ca ^2+^ oscillations.

The kinetics of the MCU and the NCLX have been fairly well characterized, but much remains to be done to fully identify other fluxes. The permeability transition pore (PTP) in its low conduction mode participates in the Ca ^2+^ exchange process in HeLa cells, as its inhibition by cyclosporine A affects Ca ^2+^ oscillations ^36, 39^. The functional role of LETM1-mediated Ca ^2+^ transport also remains poorly understood. This EF-containing transmembrane protein has been functionally identified as a Ca ^2+^/H ^+^ exchanger of the inner mitochondrial membrane ^40, 41^, although this remains controversial ^42^. In electrically excitable cells such as cardiomyocytes and neurons, ryanodine receptors have been shown to transport Ca ^2+^ into mitochondria ^43, 44^. A rapid Ca ^2+^ uptake mode (RaM) of poorly identified molecular nature has been reported in studies on isolated mitochondria from cardiac and liver cells ^45, 46^. However, the implication of RyR and RaM in mitochondrial Ca ^2+^ influx remains to be firmly established ^47, 48^. Finally, in a more indirect manner, mitochondrial metabolism also affects cytosolic Ca ^2+^ signals, mainly by modifying the mitochondrial voltage across the internal mitochondrial membrane, which greatly affects the activities of all of the above-mentioned fluxes ^29^. All of the above-cited phenomena are thus potentially implicated in the control of the detailed characteristics of Ca ^2+^ oscillations. Their interplay with the activities of the MCU and the NCLX is regulated by an intricate and complex network of interactions implicating cytosolic and mitochondrial Ca ^2+^ as well as mitochondrial voltage and numerous accessory proteins.

## Store-operated Ca ^2+^ entry

Cytosolic Ca ^2+^ oscillations are sustained by SOCE from the extracellular medium ^49^. This mechanism involves the stromal interaction molecule (STIM) and the Orai protein ^50^. The transmembrane ER protein STIM is sensitive to Ca ^2+^ changes in the ER through an EF-hand facing the lumen of the store. Decrease in luminal Ca ^2+^ below ~200 μM (for the STIM1 isoform) leads to STIM aggregation, followed by migration to ER–plasma membrane junctions. Here, STIM oligomers can bind and activate Orai, a four-transmembrane-domain plasma-membrane-spanning protein, thus forming a channel (known as CRAC for Ca ^2+^-release-activated Ca ^2+^ channel) allowing Ca ^2+^ to enter down the chemical gradient. Another STIM isoform, STIM2, has a lower affinity for ER Ca ^2+^, which allows for activation of Ca ^2+^ entry at moderate ER depletion, although at a reduced rate ^51^. Mammalian cells have genes for the three homologs Orai1, Orai2, and Orai3, and it is thought that Orai2 and/or Orai3 act as compensative types for the lack of Orai1. Orai channels are made of multiple subunits, and CRAC channel gating by STIM is best described by a Monod-Wyman-Changeux scheme in which tetramers of Orai have four STIM binding sites ^50, 52^.

Although the mechanism just described has most of the time been investigated in conditions when the Ca ^2+^ pools are emptied artificially, studies performed in a variety of cell types demonstrate that STIM expression is essential for an ensemble of physiological processes ^53^. To quote here just one recent example, in airway smooth muscle, altered expression and function of STIM/Orai proteins have been linked to pathologies including restenosis, hypertension, and atopic asthma ^54^.

The STIM-Orai pathway for Ca ^2+^ entry displays a hysteretic behavior: STIM-Orai association and dissociation do not occur at similar ER Ca ^2+^ concentrations ^55^. Although the origin and the physiological significance of this unusual behavior remains unknown, it might be related to the inactivation of SOCE-mediated Ca ^2+^ entry by cytosolic Ca ^2+^ itself, a process that has long been thought to be mediated by calmodulin ^56^ but was recently suggested to be due to a calmodulin-independent conformational change within the pore allowed by two specific Orai residues, Y80 and W76 ^57, 58^. This Ca ^2+^-induced inactivation (CDI) of SOCE allows for a modulation of Ca ^2+^ entry depending on the level of cytosolic Ca ^2+^, thus shaping the oscillations.

The key effect of STIM and Orai on the oscillatory Ca ^2+^ pattern and its downstream targets are also much documented. Through the specific ER Ca ^2+^ sensor STIM2 that has a high K _M_ for Ca ^2+^, SOCE determines the basal level of Ca ^2+^ in HeLa cells ^51^. More generally, SOCE-mediated Ca ^2+^ entry has a significant effect on Ca ^2+^ oscillations, as it can in turn affect all Ca ^2+^ exchanges between the cytoplasm and the internal stores. A less straightforward but highly interesting effect was uncovered in RBL-2H3 cells. Because the activity of the plasma membrane phosphatidylinositol 4-phosphate 5 kinase that replenishes the PIP _2_ pool is Ca ^2+^ sensitive, SOCE is necessary to avoid the rundown of the oscillations. Indeed, in the absence of SOCE, cysteinyl leukotriene type I receptor activation leads to the exhaustion of the PIP _2_ pool and hence to the disappearance of InsP _3_-induced Ca ^2+^ release from internal stores ^59^.

**Figure 4.**
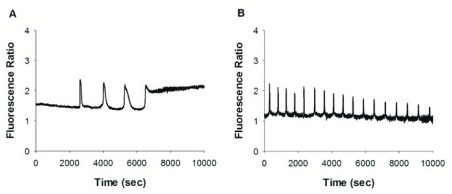
Effect of store-operated calcium entry (SOCE) activity on fertilization-induced Ca ^2+^ oscillations in pig eggs. **A.** Co-overexpression of Orai1 and stromal interaction molecule 1 (Stim1) leads to broader spikes with reduced frequency and that stop prematurely. **B.** Control situation. Reproduced with permission from Chunmin Wang, Lu Zhang, Laurie A. Jaeger, and Zoltan Machaty. Store-Operated Ca2+ Entry Sustains the Fertilization Ca ^2+^ Signal in Pig Eggs. Biol Reprod 2015; 93(1):25. DOI:10.1095/ biolreprod.114.126151 ^62^.

In RBL cells displaying Ca ^2+^ oscillating, gene expression is entirely driven by SOCE and proceeds as an all-or-nothing process in individual cells ^60^. During maturation of mouse oocytes, STIM1 and Orai1 control the basal Ca ^2+^ level and the whole Ca ^2+^ homeostasis, thus controlling meiosis resumption ^61^. At fertilization of pig eggs, overexpression of STIM1 and Orai1 substantially decreases the number of Ca ^2+^ spikes induced by sperm binding ( Figure 4). Moreover, these spikes are broader and their frequency is reduced as compared to control eggs ^62^. This observation contrasts with the observed decreased frequency of fertilization-induced Ca ^2+^ oscillations in hamster eggs when decreasing external Ca ^2+^ concentration ^63^. It shows that the control of Ca ^2+^ signaling by SOCE cannot be directly assimilated to the control of Ca ^2+^ signals by the extracellular Ca ^2+^ concentration. Interestingly, if SOCE is inhibited, fertilization is also impaired, as oscillations last for about 1 hour instead of at least 2 hours. In mice, cytoplasmic Ca ^2+^ levels are elevated for ~50% of the time in STIM1+Orai1-overexpressing oocytes in the first 2 hours after fertilization, as compared to only less than 20% of the time in control oocytes. Despite this larger Ca ^2+^ signal, most of the STIM1/Orai1-overexpressing oocytes do not reach the two-cell stage ^64^. However, female mice lacking Orai1 are fertile ^65^, while male mice are sterile due to severe defects in spermatogenesis ^66^.

## Conclusion

It is by now clear that in many cases the existence of Ca ^2+^ oscillations does not provide an ON/OFF signal for the Ca ^2+^-mediated response to the stimulus nor is the extent of the response only determined by the frequency of the oscillations. How the exact shape of this Ca ^2+^ signal is controlled, i.e. what we refer to here as its fine tuning, can alter the response qualitatively and quantitatively. Ca ^2+^ exchanges with mitochondria and SOCE play an important role in fine tuning cytosolic Ca ^2+^ oscillations. Interestingly, there is some dynamic interplay between these two Ca ^2+^ sources as, in mesothelial cells in the absence of external Ca ^2+^, mitochondrial Ca ^2+^ takes over to provide a Ca ^2+^ influx pathway during oscillations ^67^. Moreover, other organelles such as the Golgi ^68, 69^ or the acidic organelles ^21, 70, 71^ are also involved. Genetic regulations further complicate the intricate network of Ca ^2+^ fluxes: in lymphocytes, Ca ^2+^-dependent activation of CREB controls the level of expression of the MCU, which explains why the expression of this uniporter is modified in the absence of InsP _3_ receptors or of the STIM/Orai machinery ^72^. Much remains to be done to understand how diverse factors interact to control the detailed pattern of Ca ^2+^ oscillations and how this pattern can in some cases significantly affect the physiological response. Integration of the Ca ^2+^ signal over long periods of time may explain why small changes in the pattern of the Ca ^2+^ spikes become significant in some cases. By such integration, the extent of activation of the downstream targets of Ca ^2+^ is modified by apparently minor changes in the Ca ^2+^ oscillatory pattern, which are less visible than its frequency. Spatial aspects most certainly also play an important role in this respect, as the Ca ^2+^-sensitive targets are far from being homogeneously distributed within the cell ^73^. Finally, the kinetics and thresholds for Ca ^2+^ activation of these targets are expected to be at least as important, as in other signaling cascades playing a key role in the storage of information ^74^.
